# Influential publications in sudden hearing loss: a bibliometric and visual synopsis of the top 100 cited articles

**DOI:** 10.3389/fneur.2024.1494936

**Published:** 2025-01-20

**Authors:** Xueshi Di, Junjie Liang, Xinru Wang, Xue Bai, Chongyang Zhang, Ting Pan, Tiantian He, Peng Bai

**Affiliations:** ^1^The Third Affiliated Hospital of Beijing University of Chinese Medicine, Beijing, China; ^2^Beijing Hospital of Traditional Chinese Medicine, Beijing, China

**Keywords:** sudden hearing loss, bibliometrics, visual analysis, most cited articles, treatment options

## Abstract

**Background:**

Sudden hearing loss (SHL) is a prevalent emergency in otolaryngology. Despite its frequency, there is a lack of econometric analysis and visualisation of the most significant SHL research literature. This study aims to provide a comprehensive overview and explore the characteristics of the 100 most cited articles in SHL through bibliometric analysis.

**Materials and methods:**

The Web of Science Core Collection (WOSCC) was used to identify the 100 most cited SHL articles from 1999 to 2024. Tools such as CiteSpace and VOSviewer were employed to visualise data on countries, institutions, authors, co-cited authors, journals, co-cited journals, co-cited references, and keywords.

**Results:**

The citations of the 100 most cited articles ranged from 59 to 760, with publications spanning from 1999 to 2021 and peaking in 2005. The most cited article was authored by Schwartz SR. The majority of these articles originated from the United States. Key themes identified include treatment options for SHL, with prominent keywords such as deafness, therapy, and dexamethasone.

**Conclusion:**

This study identified the highly cited literature in SHL research, revealing a primary focus on treatment options. These findings provide crucial insights into the research hotspots and frontiers in the field of SHL.

## Introduction

Sudden hearing loss (SHL) is defined as an acute, idiopathic sensorineural hearing impairment characterised by a loss of at least 30 decibels (dB) across three consecutive frequencies within a 72-h timeframe (1). Patients with SHL may also present with associated symptoms including tinnitus, aural fullness, vertigo, and nausea. The exact etiology and mechanism of SHL, a common clinical otorhinolaryngological emergency, are unknown (2), Some researchers have attributed it to viral infections, circulatory system dysfunction, and immune dysfunction (3), and there is no standard treatment strategy. With the accelerating pace of life in society, the incidence of SHL is increasing year by year and tends to be younger, which has a serious impact on the quality of life of patients and may lead to psychological and mental health problems (4–6). Therefore, there is an urgent need to comprehensively analyse the complex pathogenesis of SHL and to develop evidence-based diagnostic and therapeutic solutions to address the core pathological aspects.

Bibliometric analysis is an important tool to be able to identify influential papers in a given field and to quantitatively analyse the scientometric methodology of publications (7). Articles with a high citation frequency in a given field are usually the basis of research in that field and have an important reference value, as well as providing important information about the current research situation in the field (8). Therefore, analysing the most cited articles in a field through bibliometric visualization and the use of quantitative methods can reveal the most important contributors, research bases, trends, and hotspots in the field of study.

A large number of studies on SHL have been published. However, bibliometric analyses of SHL articles with high quality and high citation frequency have not been reported, while most of the previous bibliometric analyses have been limited to analyses of countries, authors, journals, and co-cited references, and have not analysed and summarised the themes of these documents (9, 10). Considering the clinical significance of SHL and the importance of highly cited articles, we conducted qualitative and quantitative analyses of the 100 articles with the highest number of citations on SHL and innovatively analysed and summarised the themes of these literatures, to provide a comprehensive and systematic understanding of the background and current status of the research in this field or topic, and also to dig deeper into the secrets behind the literatures, to find the innovations, to predict the future development trends, etc., in order to help researchers understand the research directions and trends of SHL and make better use of classic articles on SHL for future research in this field.

## Materials and methods

### Retrieval strategies

Our study used the Web of Science Core Collection database (Index: Science Citation Index Expanded) to retrieve the top 100 most cited articles on SHL, while journals in related specialities were consulted to ensure that the literature search was as comprehensive as possible. The method was as follows: using title = “Sudden Hearing Loss” OR “Deafness, Sudden” OR “Sudden Deafness” search, search time range: 1999-01-01 to 2024-06-11, language restriction to English, literature type set to articles and reviews, and sorted in descending order of the number of times cited, which were then independently checked for eligibility by two researchers (Chongyang Zhang and Ting Pan), and if the articles had the same total number of citations, the most recent article in the year of publication was included, and ultimately the top 100 most cited eligible articles were included for subsequent analyses as plain text downloads.

### Data analysis

We used Excel to collect data related to the literature; VOSviewer (version 1.6.18) was used for the analysis of institutions, authors, co-citing authors, journals, co-citing journals, co-references and keyword view. CiteSpace (version 3.6.1) was used for keyword clustering analysis, Scimago Graphica (version 1.0.43) was used to analyse the number of national releases and collaborative views, and we searched the https://www.Letpub.com.cn website for journal impact factors and H-indexes. We read the content of these 100 papers in detail and then analysed them to summarise the topic types of these papers and combined with our analysis of keywords and co-cited references to summarise the research bases, trends, and hotspots in the field of SHL research.

## Results

### General data

**Figure 1:**
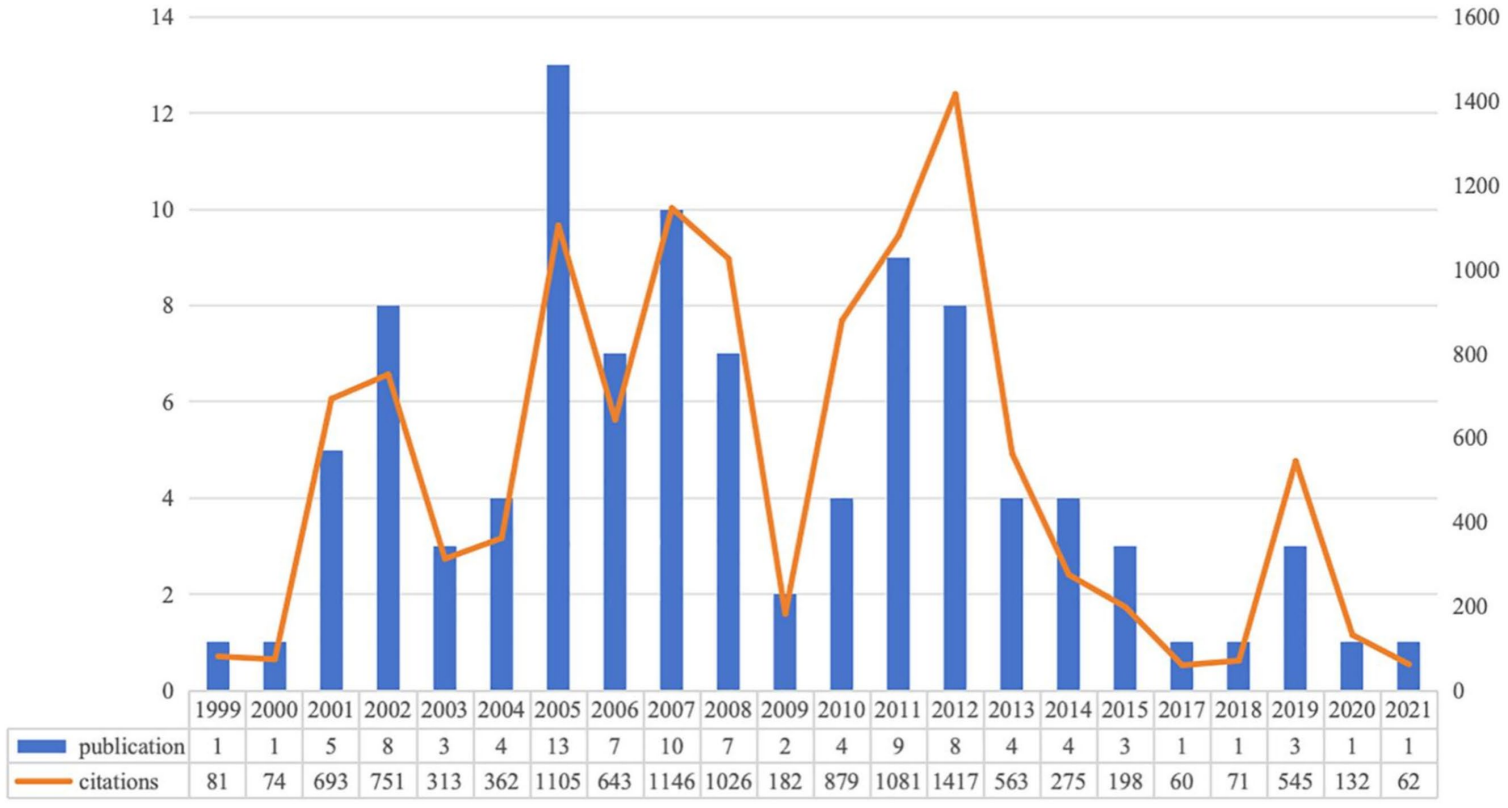
Annual distribution of publications and view of citations.

Based on our search strategy, we collected the 100 most cited articles in the field of SHL from WoSCC. The annual distribution of publications and view of the number of citations for these 100 articles, shown in Figure 1, were published between 1999 and 2021, with the highest number of articles published in 2005 (*n* = 13). These 100 articles, 88 articles, and 12 reviews were cited from 59 to 760 frequencies, and the top 10 articles are shown in Table 1.

**Table 1:** Top 10 most cited articles.

Rank	Title	Count	Journals	Year	IF	DOI
1	Clinical Practice Guideline: Sudden Hearing Loss	760	OTOLARYNGOLOGY-HEAD AND NECK SURGER	2012	3.4	10.1177/0194599812436449
2	Clinical Practice Guideline: Sudden Hearing Loss (Update)	409	OTOLARYNGOLOGY-HEAD AND NECK SURGER	2019	3.4	10.1177/0194599819859885
3	Sudden sensorineural hearing loss	348	LANCET	2010	168.9	10.1016/S0140-6736(09)62071-7
4	Systematic Review of the Evidence for the Etiology of Adult Sudden Sensorineural Hearing Loss	314	LARYNGOSCOPE	2010	2.6	10.1002/lary.20873
5	Sudden Sensorineural Hearing Loss: A Review of Diagnosis, Treatment, and Prognosis	307	TRENDS IN AMPLIFICATION	2011	–	10.1177/1084713811408349
6	Idiopathic sudden sensorineural hearing loss	250	NEW ENGLAND JOURNAL OF MEDICINE	2008	158.5	10.1056/NEJMcp0802129
7	Incidence of Sudden Sensorineural Hearing Loss	249	OTOLOGY & NEUROTOLOGY	2013	2.1	10.1097/MAO.0000000000000222
8	Oral vs. Intratympanic Corticosteroid Therapy for Idiopathic Sudden Sensorineural Hearing Loss A Randomized Trial	244	JAMA-JOURNAL OF THE AMERICAN MEDICAL ASSOCIATION	2011	120.7	10.1001/jama.2011.679
9	Intratympanic dexamethasone for sudden sensorineural hearing loss: Clinical and laboratory evaluation	239	OTOLOGY & NEUROTOLOGY	2001	2.1	10.1097/00129492-200101000-00005
10	Intratympanic dexamethasone for sudden sensorineural hearing loss after failure of systemic therapy	217	LARYNGOSCOPE	2007	2.6	10.1097/01.mlg.0000245058.11866.15

### Countries and institutions

**Figure 2:**
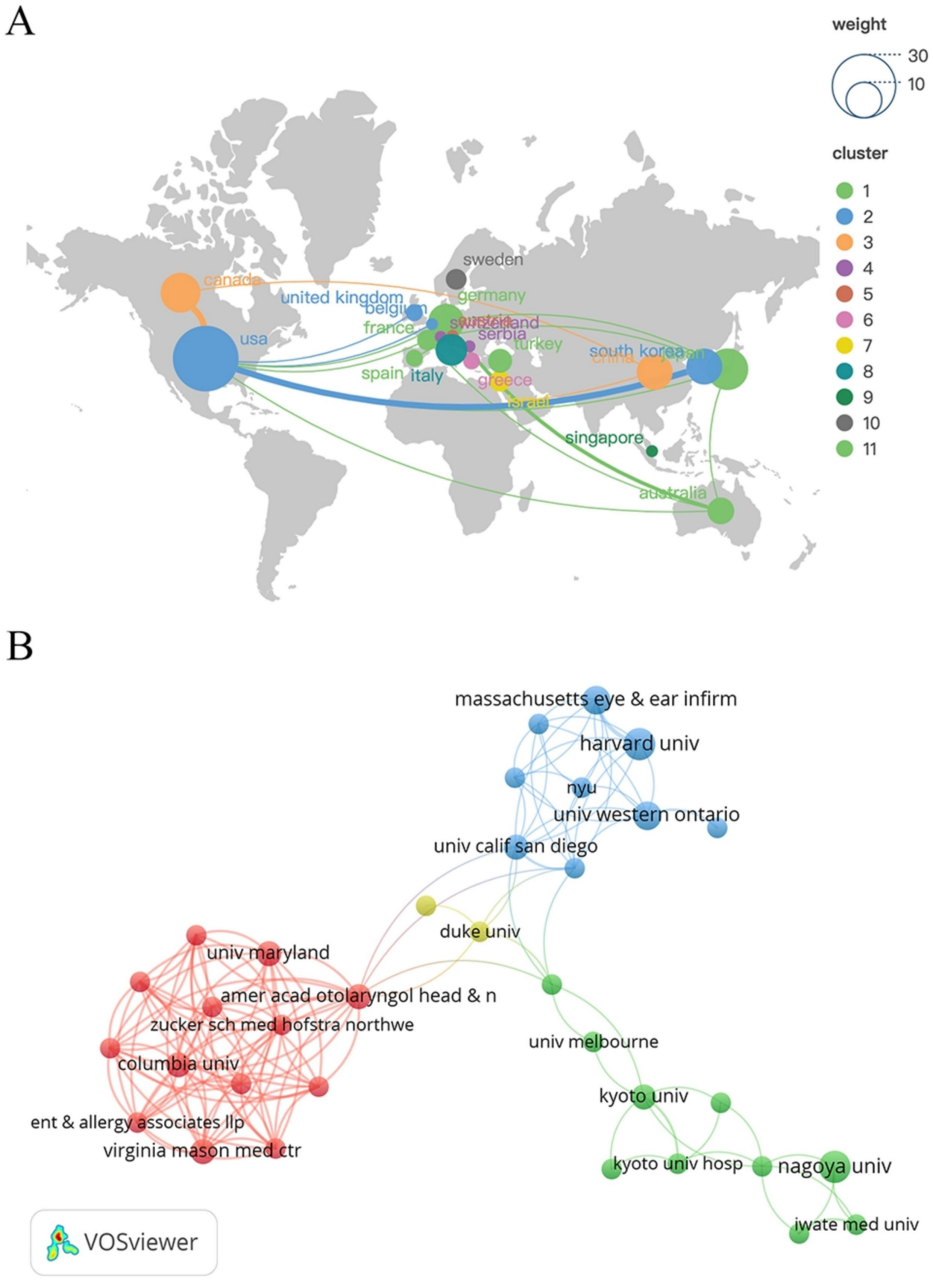
View of national **(A)** and institutional **(B)** collaborative networks in the field of SHL research.

These 100 articles were published by 20 countries with the participation of 194 institutions. The top 10 countries in terms of the number of articles are shown in Table 2, and the country with the highest number of articles is the United States, followed by Japan and Canada. Using VOSviewer software to analyse the network view of these 20 countries, as shown in Figure 2A, which demonstrates the cooperation relationship between the countries, it can be seen that the United States and Canada have close co-operation, in addition, the United States and South Korea, Germany, and Australia also have close co-operation, but the co-operation exchanges between other countries are weak. The top 10 institutions in terms of the number of articles are shown in Table 2, which shows that seven of these 10 institutions are from the United States, and the institutions with the largest number of articles are from the United States and Japan, indicating that the United States and Japan have more comprehensive and in-depth research in this field. As shown in Figure 2B, which demonstrates the collaboration between the 49 institutions with an article volume greater than or equal to 2, it can be seen that Harvard University and Massachusetts Eye and Ear Infirmary have a close collaboration.

**Table 2:** Top 10 countries and institutions in terms of number of articles issued.

Rank	Country	Publications	Citations	Institution	Publications	Citations
1	United States	30	4,863	Harvard University	5	937
2	Japan	12	921	Nagoya University	5	337
3	Canada	11	1,785	Massachusetts Eye and Ear Infirmary	4	687
4	Germany	9	818	University of Western Ontario	4	662
5	South Korea	9	756	American Academy of Otolaryngology–Head and Neck Surgery Foundation	3	1,242
6	China	9	861	Columbia University	3	544
7	Italy	7	658	University of Maryland	3	584
8	Australia	5	475	Virginia Mason Medical Center	3	572
9	Turkey	4	327	University of California, SanDiego	3	1,253
10	France	3	271	Keimyung University	3	284

### Authors and co-citing authors

**Figure 3:**
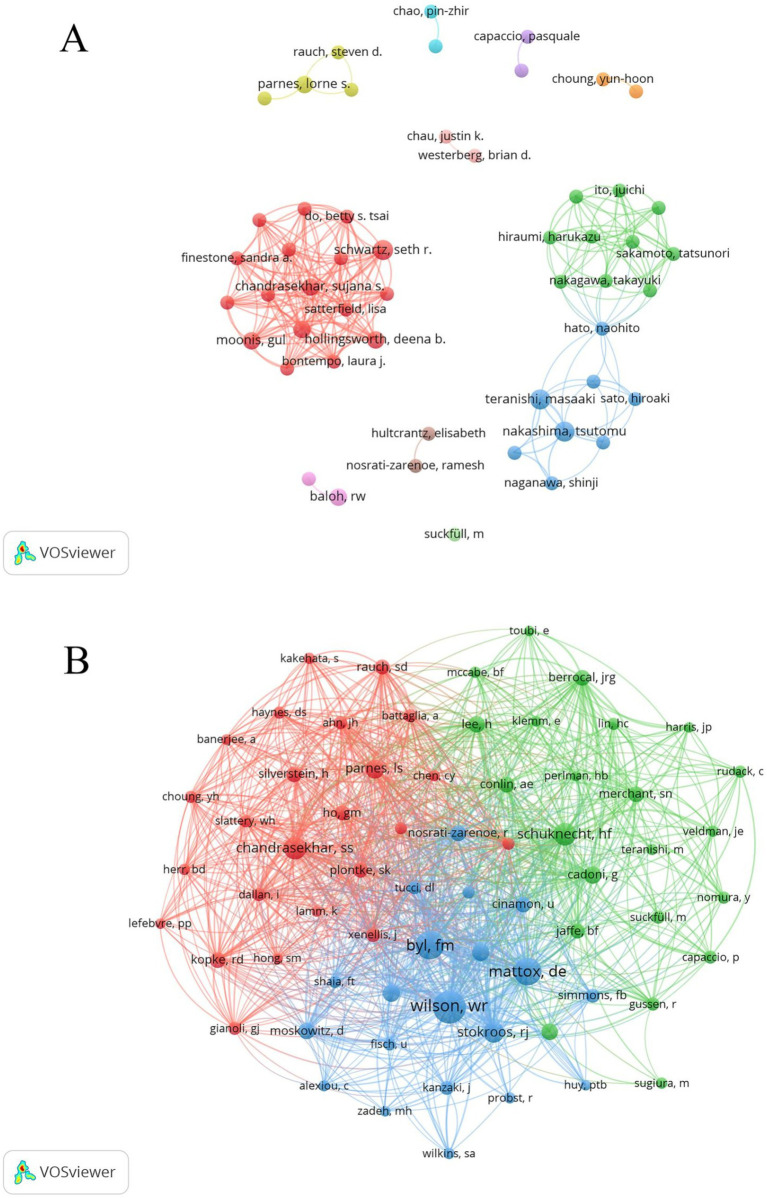
Network view of authors **(A)** and co-citing authors **(B)**.

A total of 476 authors were involved in the publication of these 100 articles. The top 10 authors in terms of the number of publications are shown in Table 3, and the authors with the highest number of publications are Schwartz SR, Nakashima T, and Teranishi M. Schwartz SR’s articles have the highest number of citations among the top 10 authors, which indicates that he has a high reputation in the field of research and deserves to be learned from by the researchers. Using VOSviewer software to analyse the collaborative view of the 52 authors with more than or equal to two publications, as shown in Figure 3A, it can be seen that individual authors do not collaborate too closely with each other. Our analysis revealed that a total of 1,453 authors were listed among the co-cited authors in this research area, with seven authors being cited more than 30 times, Wilson WR (92) being the most cited, followed by Byl FM (69), and Mattox DE (66). The 66 authors with equal to or more than 10 co-citations were analysed in a view using VOSviewer software, as shown in Figure 3B, and were divided into three clusters represented by three colorsors, namely Chandrasekhar SS and Parnes LS centred on the red cluster, Wilson WR, Byl FM and Mattox DE centred on the blue cluster, and Schuknecht HF and Cadoni G centred on the green cluster.

**Table 3:** Top 10 authors in terms of number of publications and co-citations.

Rank	Authors	Publications	Citations	Co-authors	Citations
1	Schwartz, SR	4	1,332	Wilson, WR	92
2	Nakashima, T	4	313	Byl, FM	69
3	Teranishi, M	4	313	Mattox, DE	66
4	Chandrasekhar, SS	3	1,242	Schuknecht, HF	44
5	Hollingsworth, DB	3	1,242	Chandrasekhar, SS	43
6	Stachler, RJ	3	1,242	Stokroos, RJ	39
7	Moonis, G	3	544	Parnes, LS	33
8	Parnes, LS	3	581	Fetterman, BL	28
9	Baloh, RW	3	284	Hughes, GB	27
10	Bontempo, LJ	2	482	Cadoni, G	26

### Journals and co-cited journals

**Figure 4:**
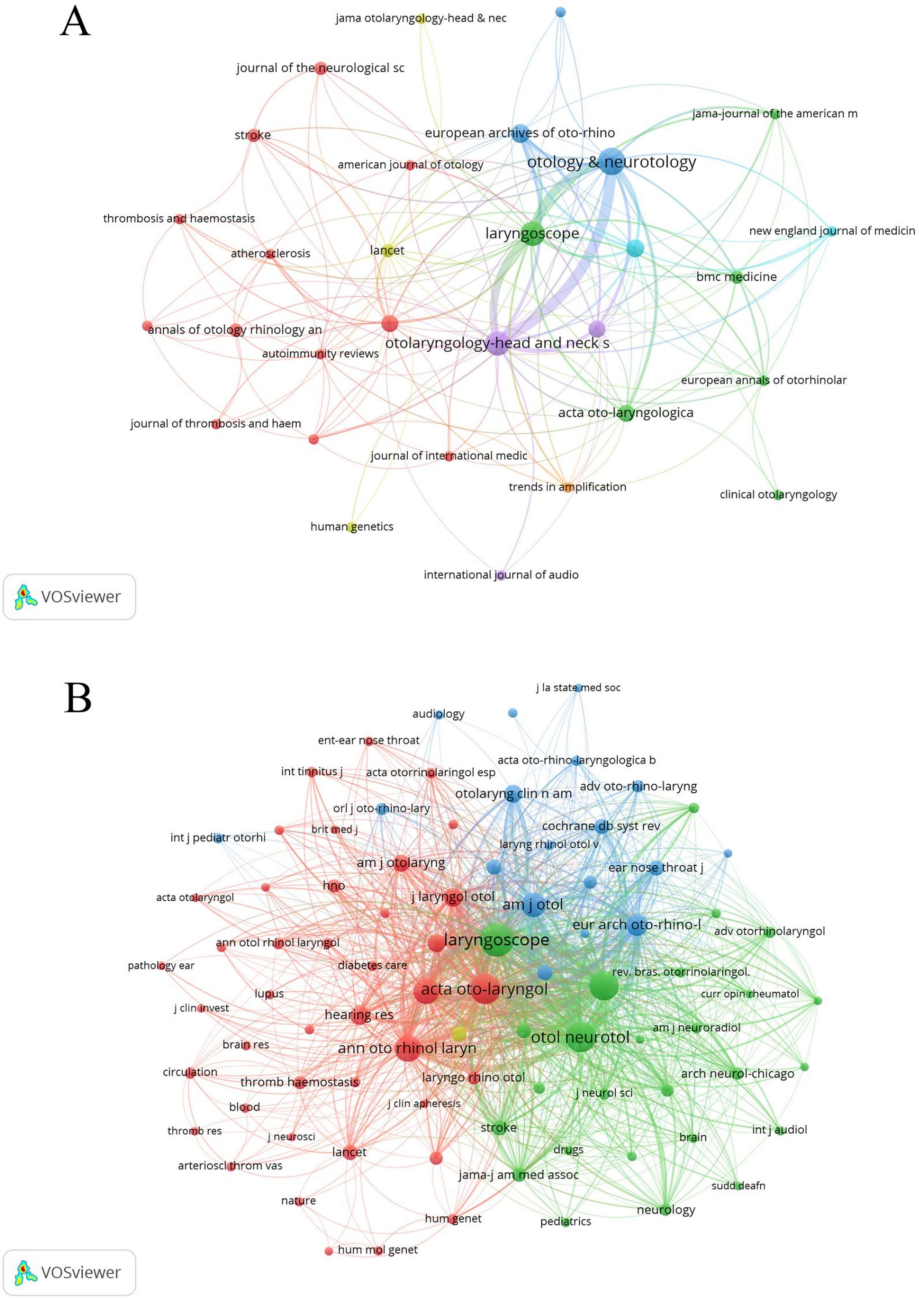
Visualisation of journals **(A)** and co-cited journals **(B)**.

We analysed that 100 articles of this study were published in 31 journals and co-cited in 610 journals; the top five journals in terms of the number of journal articles and co-cited journals are shown in Table 4. OTOLOGY & NEUROTOLOGY published the highest number of articles with 21 articles, followed by OTOLARYNGOLOGY-HEAD AND NECK SURGERY (14) and LARYNGOSCOPE (14). By view analysis, as shown in Figure 4A, there is a positive citation relationship between OTOLOGY & NEUROTOLOGY, OTOLARYNGOLOGY-HEAD AND NECK SURGERY, and LARYNGOSCOPE; and co-cited journals view, as shown in Figure 4B, a total of 86 journals were cited more than 5 times. The top three co-cited journals are LARYNGOSCOPE, OTOLOGY & NEUROTOLOGY, and ACTA OTO-LARYNGOLOGICA, and these are journals with considerable impact in the ENT research direction; furthermore, as can be seen from the figure, the top 5 co-cited journals have positive co-citation relationships.

**Table 4:** Top 5 journals and co-cited journals.

Rank	Journals	Count	IF	H-index	Co-cited journal	Co-citation	IF	H-index
1	OTOLOGY & NEUROTOLOGY	21	1.9	93	LARYNGOSCOPE	447	2.2	134
2	OTOLARYNGOLOGY-HEAD AND NECK SURGERY	14	2.6	109	OTOLOGY & NEUROTOLOGY	293	1.9	93
3	LARYNGOSCOPE	14	2.2	134	ACTA OTO-LARYNGOLOGICA	290	1.2	72
4	EUROPEAN ARCHIVES OF OTO-RHINO-LARYNGOLOGY	6	1.9	61	OTOLARYNGOLOGY-HEAD AND NECK SURGERY	285	2.6	109
5	ARCHIVES OF OTOLARYNGOLOGY-HEAD & NECK SURGERY	5	2.3	2.3	ANNALS OF OTOLOGY RHINOLOGY AND LARYNGOLOGY	195	1.3	81

### Commonly cited references

We applied VOSviewer software to analyse these 100 articles for co-cited references and found that these 100 documents co-cited 1,886 articles, with 41 articles co-cited more than 10 times, and screened out the top 5 references for co-citation frequency, as shown in Table 5.

**Table 5:** Five documents with the highest frequency of co-cited references.

Rank	Co-cited reference	Citations
1	Wilson WR, Byl FM, Laird N. The efficacy of steroids in the treatment of idiopathic sudden hearing loss. A double-blind clinical study. Arch Otolaryngol. 1980 Dec;106 (12):772–6. doi: 10.1001/archotol.1980.00790360050013.	58
2	Byl FM Jr. Sudden hearing loss: eight years’ experience and suggested prognostic table. Laryngoscope. 1984 May;94 (5 Pt 1):647–61. PMID: 6325838.	58
3	Mattox DE, Simmons FB. Natural history of sudden sensorineural hearing loss. Ann Otol Rhinol Laryngol. 1977 Jul-Aug;86 (4 Pt 1):463–80. doi: 10.1177/000348947708600406.	48
4	Parnes LS, Sun AH, Freeman DJ. Corticosteroid pharmacokinetics in the inner ear fluids: an animal study followed by clinical application. Laryngoscope. 1999 Jul;109 (7 Pt 2):1–17. doi: 10.1097/00005537-199907001-00001.	33
5	Chandrasekhar SS. Intratympanic dexamethasone for sudden sensorineural hearing loss: clinical and laboratory evaluation. Otol Neurotol. 2001 Jan;22 (1):18–23. doi: 10.1097/00129492-200101000-00005.	25

### Keyword analysis

**Figure 5:**
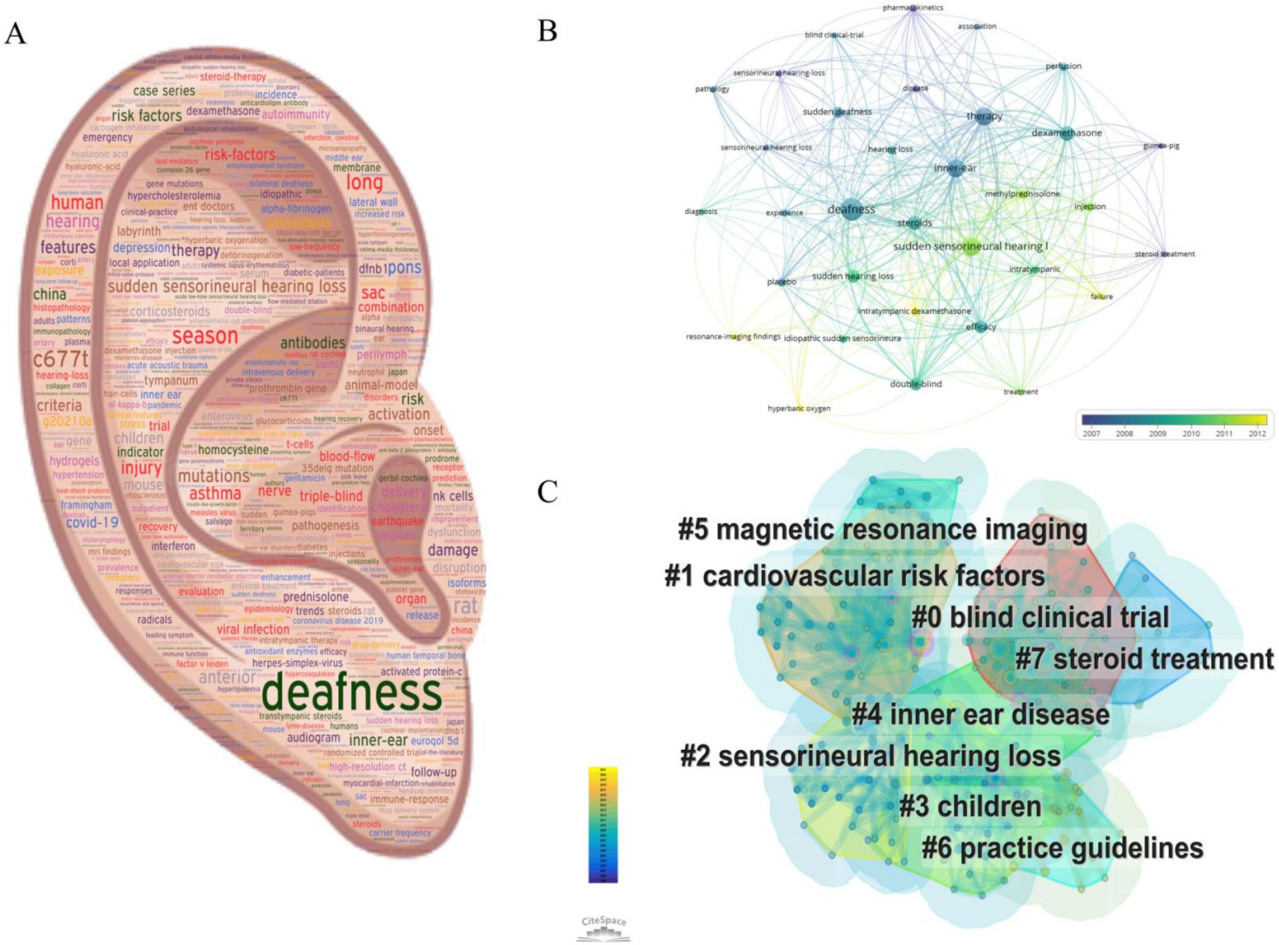
**(A)** Keyword word cloud view; **(B)** Overlay visualisation of keyword frequency trends over time; **(C)** Keyword cluster analysis mapping.

We used VOSviewer software to do keyword analysis on these 100 documents and came up with 399 keywords, 33 keywords with a frequency of occurrence greater than or equal to 5, and the top 20 keywords with the highest frequency of occurrence are shown in Table 6. In https://wordart.com, word cloud analysis for 399 keywords, as shown in Figure 5A, the word size in the word cloud graph represents the frequency of occurrence. In the VOSviewer software, to construct a superimposed visual graph analysis of the time trend of the screened 33 keywords, as shown in Figure 5B, it can be seen that hyperbaric oxygen, residual-imaging findings, and intratympanic dexamethasone are the high-frequency words in the recent years.

**Table 6:** Top 20 most frequent keywords.

Rank	Keyword	Counts	Rank	Keyword	Counts
1	deafness	43	11	methylprednisolone	9
2	sudden sensorineural hearing loss	31	12	injection	9
3	therapy	29	13	placebo	9
4	inner-ear	29	14	intratympanic	8
5	dexamethasone	21	15	perfusion	8
6	steroids	19	16	intratympanic dexamethasone	8
7	double-blind	17	17	hearing loss	8
8	efficacy	16	18	idiopathic sudden sensorineural hearing loss	8
9	sudden hearing loss	16	19	pharmacokinetics	7
10	sudden deafness	12	20	experience	7

Keyword clustering analysis responds to the research hotspots and trends in the research field. We used CiteSpace software to analyse the keyword clustering of this study, and the results are shown in Table 7 and Figure 5C. The first six keyword clusters are blind clinical trial, inner ear disease, cardiovascular risk factors, children, and magnetic resonance imaging.

**Table 7:** Top 6 clusters in the keyword co-occurrence map.

ClusterID	Size	Silhouette	Year	Label (LLR)
#0	60	0.808	2008	blind clinical trial
#1	38	0.946	2007	cardiovascular risk factors
#2	31	0.841	2005	sensorineural hearing loss
#3	27	0.791	2007	children
#4	21	0.863	2006	inner ear disease
#5	20	0.916	2010	magnetic resonance imaging

### Theme analysis of literature

**Figure 6:**
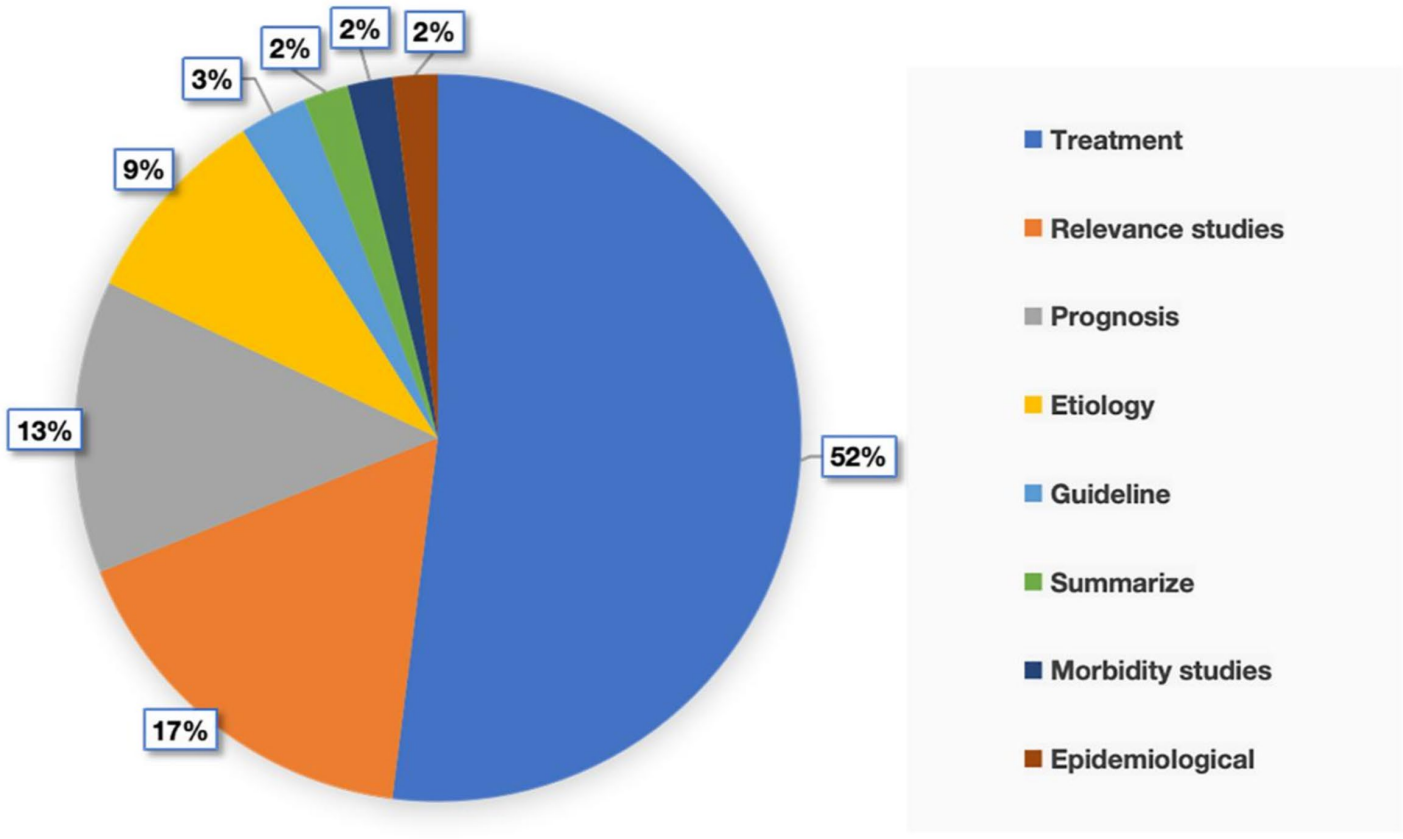
Types of literature themes.

After we read the contents of these 100 literatures in detail, we summarised the themes of these literatures and came up with the following eight themes: (1) research on the treatment of SHL; (2) research on the association between SHL and other diseases; (3) research on the prognosis of SHL; (4) research on the etiology of SHL; (5) clinical guidelines on SHL; (6) reviews on SHL; (7) research on the incidence of SHL and (8) research on the epidemiology of SHL. The percentage of the number of each topic type in these 100 publications is shown in Figure 6, which clearly shows that more than half of the research publications on the treatment of SHL, indicating that this type of research topic is the main and hot topic of SHL

## Discussion

According to our collection of the top 100 most cited articles in the field of SHL from WoSCC, which were published between 1999 and 2024, the total citation frequency of articles published in the last 3 years did not make it into the top 100, suggesting that a period of exposure is needed for recently published papers to increase citations. While it is true that the number of citations a paper generates is an indicator of the impact of a field of research, it does not necessarily mean that it is a symbol of the high quality and prominence of that field of research (11). The number of citations in the literature accumulates over time, and therefore analysing citation strength readings through time affects the assessment, as Garfield demonstrates by showing that earlier papers have a greater chance of being cited (12), its argued that even the most highly cited papers were not cited when they were first published were not cited. The number of citations for these 100 publications in this study ranged from 59 to 760, with the highest number of single publications in 2005 (13) and 2007 (10), which shows that the field of SHL research has received more attention in these 2 years, which may be due to the increase in the prevalence of SHL globally.

The most cited literature in our study was the Clinical Practice Guideline on Sudden Hearing Loss published in 2012 (13), which provides evidence-based recommendations for the diagnosis, management, and follow-up of patients with SHL and focuses primarily on sudden sensorineural hearing loss (SSNHL) in adult patients (18 years of age and older), which provides important This provides important guidance to ENT practitioners and researchers. The second most cited document was an update to the Clinical Practice Guideline on Sudden Hearing Loss published in 2012 (1), the purpose of this guideline update is to provide clinicians with evidence-based recommendations for evaluating the diagnosis, management, and follow-up of patients with sudden deafness and sudden sensorineural deafness. The third most cited document was a review paper on SSNHL published in the high-quality journal Lancet (2), which provides an overview of the clinical presentation, etiology, assessment, prognosis, and treatment of SSNHL, offering a practical approach to managing this complex and poorly understood patient population. These guidelines mentioned above, while reflecting their safety and efficacy, are more than a decade old, suggesting that more attempts are needed to promote their development. The topics of the remaining seven papers in the top 10 most cited papers focused on the study of treatment, etiology, and incidence of SHL, which shows that these lines of research are the current basis of research and concern in this field of study.

Based on the country perspective on the number of publications, the United States, Japan and Canada are the three countries with the highest number of publications, and the United States has the highest number of publications, which is far more than the other countries, indicating that the United States has invested a large amount of funds and manpower in the research of SHL. From the view of cooperation between countries, the United States has close cooperation with Canada and South Korea, and Germany and Australia also have close cooperation, but the cooperation between other countries is not close enough, which is obviously not conducive to the exchange and cooperation in this research field and also not conducive to the development of this research field. Therefore, the exchange and co-operation between countries should be strengthened in order to promote the development of this research field in the future. In terms of the number of articles published by institutions, most of the top 10 institutions are from the United States, which indicates that the research on SHL is highly valued by institutions in the United States; however, as can be seen from the chart of institutional cooperation, the cooperation among institutions still needs to be improved and strengthened.

From the perspective of author publications, Schwartz SR, Nakashima T, and Teranishi M are the three authors with the most publications. Schwartz SR was primarily involved in the 2012 Clinical Practice Guideline on Sudden Hearing Loss (13) and the 2019 update of this guideline (1) and the executive summary (14), he was also involved in a systematic evaluation of the existing literature on SHL to determine the efficacy of intra-drum steroid treatment for idiopathic sudden sensorineural deafness (15). Nakashima T and Teranishi M were jointly involved in the study of three-dimensional fluid-attenuated inversion-recovery magnetic resonance imaging findings and prognosis of sudden sensorineural deafness (16, 17), they also investigated the 30-year trend of SHL in four national epidemiological surveys in Japan, which yielded that the number of patients with SHL both the number of patients and the mean age, has increased (18); Furthermore, Nakashima T investigated the long-term outcome and the incidence of recurrence in patients with idiopathic sudden sensorineural deafness, concluding that recurrence of sudden deafness is very rare, and that although some patients showed significant further deterioration of hearing, the degree of deterioration of hearing in the affected versus non-affected side of the body was not significantly different in almost all patients difference (19). The above authors’ study provides a good accumulation of research on SHL and provides an important reference value for subsequent research scholars. In terms of co-citing authors of this study, Wilson WR, Byl FM and Mattox DE are the three most cited authors. Wilson WR published early research papers on SHL from 1980 to 1994, focusing on the viral factors and epidemiology of idiopathic SHL (20), he also predicted the recovery of idiopathic sudden hearing loss associated factors, discussed the interrelationships between prognostic factors and their relative importance in predicting hearing recovery (21), it also investigated the relationship between idiopathic sudden hearing loss and diabetes mellitus (22), the use of double-blind clinical studies to investigate the efficacy of steroids in the treatment of idiopathic sudden hearing loss (23), this has brought an important reference value to the subsequent study of SHL. Byl FM focused on the etiology of SHL, morbidity, treatment and prognosis (24–27). While Mattox DE has done extensive in-depth studies on the etiology, clinical staging, and treatment options of SHL (28–30). All the above-mentioned authors have done in-depth studies in the field of SHL research with high academic impact, which provide valuable citation reference value for the subsequent studies.

We found that of our 100 literatures, although the 100 most cited articles appeared in a total of 31 different journals, five journals, OTOLOGY & NEUROTOLOGY, OTOLARYNGOLOGY-HEAD AND NECK SURGERY, LARYNGOSCOPE, EUROPEAN ARCHIVES OF OTO-RHINO-LARYNGOLOGY and ARCHIVES OF OTOLARYNGOLOGY-HEAD & NECK SURGERY were the main sources for more than half of the articles (*n* = 60). We have found that these papers essentially cover the field and that these journals and co-induced journals are relatively top-tier journals specialising in the field of ear, nose, and throat research. Thus, reading these specialised and top journals can help professional otolaryngologists to quickly access the latest information and research base in the field.

Co-cited references are references that are cited together in the field of research and can be considered as the basis of research in that field. When analysing the co-cited references in this study, it was found that three out of the five articles with the highest frequency of co-citation were in the direction of research on the treatment of SHL, which shows that this direction of research is a hotspot and a trend in the field of research and is the basis of the research on the relevance of the treatment of SHL. Wilson WR et al. 1980 published the most co-cited article (23), which was a double-blind clinical study of the efficacy of steroids in the treatment of idiopathic sudden hearing loss, and found that steroids had a significant effect on hearing recovery in patients with moderate hearing loss. The hypothesis that viral cochleitis is the main cause of idiopathic sudden hearing loss; and Parnes LS et al. 1999 did an animal study and a clinical application study on the pharmacokinetics of corticosteroids in the inner ear fluid (31), establishing cochlear fluid pharmacokinetic profiles of hydrocortisone, methylprednisolone, and dexamethasone in the guinea pig following oral, intravenous, and topical (intratympanic) administration, and findings demonstrated a much higher penetration of all three drugs into the cochlear fluids following topical application as compared with systemic administration, with methylprednisolone showing the best profile, providing researchers with support for clinical applications and basic experimental studies. Meanwhile, a clinical and laboratory evaluation of intracameral dexamethasone for the treatment of sudden sensorineural deafness (SSNHL) by Chandrasekhar SS et al. 2001 (32) also found that intracameral infusion of dexamethasone significantly improves hearing as compared to intravenous dexamethasone injection Dexamethasone significantly improved hearing and had significantly higher ectolymphatic concentrations of the steroid, suggesting that intracameral instillation of dexamethasone in the tympanic cavity is a suitable therapeutic option for the treatment of SSNHL, but further studies on the dose and frequency of administration are needed. In addition, the other two were prospective studies of SHL, analysing its etiology, incidence, acute and late prognosis, and treatment options (26, 30). In conclusion, the co-cited references underpin the field of this study and provide strong evidence and guidance for future research.

Keyword analysis can grasp the core content and frontiers of a research field (33), which is helpful for us to quickly capture the distribution and development of hotspots in the field of SHL research. In this study, the top 20 keywords mainly included sudden sensorineural hearing loss, treatment, drugs, and drug delivery modes of SHL. Excluding the topic words of this study, based on the keyword analysis, we summarised and identified the most important research hotspots in these 100 literatures in two aspects, including (1) the study of sudden sensorineural hearing loss in SHL (2, 34–36), which can be seen to be the current focus of this field of research, and (2) studies on treatment options for SHL, mainly focusing on the efficacy and mode of administration of dexamethasone, methylprednisolone, and steroids (37–41).

Based on the cluster analysis of keywords, we concluded that blind clinical trial, cardiovascular risk factors, sensorineural hearing loss, children, inner ear disease and magnetic resonance imaging are emerging trends and trends for future research. Based on the analysis of the results of the study, we believe that the current direction of progress in the study of SHL mainly includes the following six aspects: (1) blind clinical trial: the treatment options for SHL are currently underway, and a large number of blind clinical trials are needed to validate the exact options, such as effective medications and routes of administration (42–45); (2) cardiovascular risk factors: cardiovascular risk factors are closely associated with the incidence, severity and prognosis of SHL, and are being studied by a large number of scholars (46–49); (3) sensorineural hearing loss: Sensorineural hearing loss is the main type of hearing loss, mainly due to inner ear damage and various etiologies such as ischemia, noise, trauma, ageing, and ototoxic medications, and is the current focus of this research area, which is currently focusing on therapeutic targets, protocols, and prognosis (50–52); (4) children: SHL in the paediatric population is a rare phenomenon, but it has significant detrimental effects on language learning and social development, and there is a need to determine the etiology, clinical signs, treatment, and management options for this disease in the paediatric population (53–55); (5) inner ear disease: Sudden sensorineural deafness (SSNHL), Meniere’s disease (MD), and autoimmune inner ear disease (AIED) are just some of the areas where researchers in the field of otorhinolaryngology are making waves (56, 57); (6) magnetic resonance imaging: Magnetic resonance imaging (MRI) can provide key measurement parameters of important value for the diagnosis and prognosis of SHL (58–60), which deserves in-depth study by scholars. In summary, these keywords in the field of SHL research reflect the current trends in this research field and will become hot spots for future research.

According to our detailed reading of the contents of these 100 literatures and analysis of the types of research topics, we found that most of the research topics mainly focus on the research of the treatment of SHL, which shows that the research in the direction of treatment is the hot spot and trend in this research field. By reviewing the relevant literature, we found that current research on treatment options for SHL has focused on treatments including *ginkgo biloba* extract (3), dexamethasone (61), steroids (62, 63), and hyperbaric oxygen (64, 65). At the same time, the correlation between SHL and other diseases is also a theme in this research field, which is being researched by a large number of scholars, such as the study of the correlation between SHL and cardiovascular diseases (66, 67). In addition, the SHL prognosis and prognosis-related factors have also received attention (68–70). These research theme analyses will provide reliable data bases and future research directions for subsequent research scholars.

It goes without saying that our bibliometric analysis has certain shortcomings. First, this analysis may have missed pertinent studies because it just used the WoSCC database, ignoring other databases. Second, we only looked at articles written in English, which might not include all research in the area. Lastly, the publications’ citation rates are always changing, which could impair the data analysis’s findings.

## Conclusion

This study reveals the top 100 highly cited publications in SHL research, demonstrates the important foundations of SHL, and identifies influential authors, institutions, countries, and journals that have made outstanding contributions to the field. Overall, the United States, as the birthplace of SHL, is the most researched, influential, and has made the most prominent contributions to the development of SHL, and with the research themes of these 100 publications focusing on the study of treatment options for SHL, the insights from this study into research priorities and trends can aid in future scholarly pursuits and provide important and valuable information for exploring future research directions.

## Data Availability

The original contributions presented in the study are included in the article/supplementary material, further inquiries can be directed to the corresponding author.
